# Inhibiting the NLRP3 Inflammasome

**DOI:** 10.3390/molecules25235533

**Published:** 2020-11-25

**Authors:** Lina Y. El-Sharkawy, David Brough, Sally Freeman

**Affiliations:** 1 Division of Pharmacy and Optometry, School of Health Sciences, Faculty of Biology, Medicine and Health, Manchester Academic Health Science Centre, The University of Manchester, Stopford Building, Oxford Road, Manchester M13 9PT, UK; lina.el-sharkawy@postgrad.manchester.ac.uk; 2 Division of Neuroscience and Experimental Psychology, School of Biological Sciences, Faculty of Biology, Medicine and Health, Manchester Academic Health Science Centre, The University of Manchester, AV Hill Building, Oxford Road, Manchester M13 9PT, UK; David.Brough@manchester.ac.uk

**Keywords:** inflammation, NLRP3, inflammasome, cytokine, cryo-EM, drug discovery, medicinal chemistry

## Abstract

Inflammasomes are protein complexes which are important in several inflammatory diseases. Inflammasomes form part of the innate immune system that triggers the activation of inflammatory cytokines interleukin (IL)-1β and IL-18. The inflammasome most studied in sterile inflammation and non-communicable disease is the NLRP3 inflammasome. Upon activation by diverse pathogen or disease associated signals, NLRP3 nucleates the oligomerization of an adaptor protein ASC forming a platform (the inflammasome) for the recruitment and activation of the protease caspase-1. Active caspase-1 catalyzes the processing and release of IL-1β and IL-18, and via cleavage of the pore forming protein gasdermin D can drive pyroptotic cell death. This review focuses on the structural basis and mechanism for NLRP3 inflammasome signaling in the context of drug design, providing chemical structures, activities, and clinical potential of direct inflammasome inhibitors. A cryo-EM structure of NLRP3 bound to NEK7 protein provides structural insight and aids in the discovery of novel NLRP3 inhibitors utilizing ligand-based or structure-based approaches.

## 1. Introduction

Since our 2015 review titled “Inhibiting the Inflammasome: A Chemical Perspective” [1] there have been substantial developments in inflammasome biology and chemistry, with inflammasome inhibitors being tested in preclinical models for the treatment of several inflammatory disorders and entering clinical trials [2,3,4,5,6]. This review focuses on the design and development of small molecule inflammasome inhibitors, with attention on the mechanism of direct NLRP3 (NACHT, LRR, and PYD Domains-Containing Protein 3) inhibitors. We also discuss the potential of the published cryo-EM structure of NLRP3 bound to NEK7 (NIMA Related Kinase 7) [7] to further develop direct small molecule inhibitors of the inflammasome.

## 2. Inflammasomes and Innate Immunity

Inflammation is generally regarded as a protective mechanism in case of infection or injury by the removal of toxins by immune cells. Non-sterile inflammation occurs from either parasites, fungi, viruses or bacteria. Sterile inflammation results from injury, foreign bodies, or hypersensitivity. Under normal conditions, immune cells are found circulating in the blood. However, upon recognition of toxins by host cells, immune cells, and their components are recruited to remove the damaging agents and promote tissue repair. Receptors that recognize pathogen-associated molecular patterns (PAMPs) and damage associated molecular patterns (DAMPs) are located on innate immune cells and are termed pattern recognition receptors (PRRs). NOD-Like receptors (NLRs) are cytosolic PRRs detecting both PAMP and DAMP signals. Some NLRs form an inflammatory complex called an inflammasome [8]. This review focuses on the inhibition of the inflammasome formed by the NLR NLRP3, a key protein involved in sterile inflammation. NLRP3 is a drug target of significant interest to the pharmaceutical industry, with potential for the treatment of several inflammatory diseases including cancer [9], autoimmune diseases [10], metabolic disorders [11], cardiovascular disease [12], CNS diseases [13], and infectious diseases including COVID-19 [14].

## 3. The NLRP3 Inflammasome

**Figure 1:**
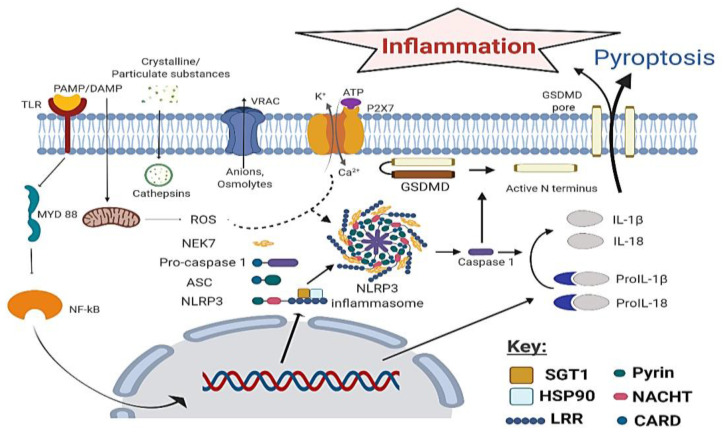
Priming and secondary signals for NLRP3 activation and its role in inflammation. Primary signal includes activation of Nuclear Factor kappa-light-chain-enhancer of activated B cells (NF-κB) mediated by myeloid differentiation primary response 88 (MYD88) by binding of P pathogen-associated molecular patterns (PAMPs) and damage associated molecular patterns (DAMPs) to TLR, and its translocation to the nucleus to undergo transcription and translation of NLRP3 inflammasome components as well as pro-inflammatory cytokines. Secondary signals which result in NLRP3 inflammasome activation include ROS from mitochondrial disruption, cathepsins, VRAC efflux of anions and osmolytes and Ca^2+^ influx. Oligomerization and activation of NLRP3 inflammasome results in cleavage of pro-inflammatory cytokines proIL-1β and proIL-18 through the action of caspase-1 to give the mature forms inflammatory cytokines interleukin (IL)-1β and IL-18, which cause inflammation. This figure was created under license using BioRender.com. https://biorender.com/.

The activation of the canonical NLRP3 inflammasome pathway requires two stages. The first is the priming stage which involves myeloid differentiation primary response 88 (MYD88), and Nuclear Factor kappa-light-chain-enhancer of activated B cells (NF-κB) signaling responsible for the expression of NLRP3 and pro-IL-1β [15]. Under normal conditions the leucine-rich repeat (LRR) maintains the NLRP3 in an auto-repressed state [15]. However, in response to a second PAMP or DAMP stimulus NLRP3 becomes active in the second stage. The pyrin domain (PYD) of active NLRP3 now binds to the PYD domain of ASC (apoptosis-associated speck-like protein containing a CARD (caspase recruitment domain)) resulting in a helical assembly of ASC to form the ASC speck/pyroptosome. Afterwards, CARD-CARD interaction between ASC and procaspase-1 takes place. Procaspase-1 yields caspase-1 via its own proteolytic cleavage which subsequently catalyzes the cleavage of pro-IL-1β and pro-IL-18 to their active forms which cause inflammation (Figure 1) [16]. Caspase-1 also leads to a type of programmed cell death known as pyroptosis resulting from gasdermin D (GSDMD) cleavage. GSDMD binds to lipids in the cell membrane resulting in pore formation, with cellular swelling and rupture releasing inflammatory cytokines IL-1β and IL-18 (Figure 1) [17].

## 4. Structure of NLRP3 and Cryo-EM Structure of NLRP3 Bound to NEK7

**Figure 2:**
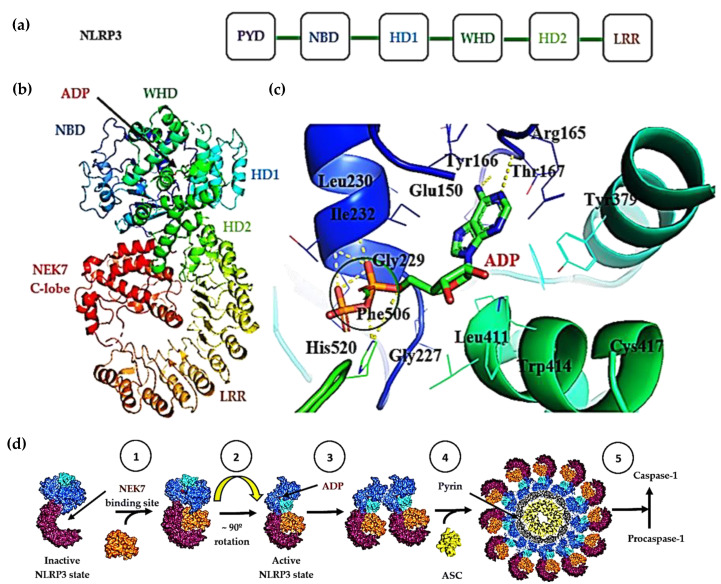
(**a**) Structural domains of NLRP3; (**b**) cryo-EM structure of NLRP3 with bound ADP (green) (PDB: 6NPY) [7] focusing on the active site of NLRP3 with ADP bound in the NACHT domain (ribbon diagram); (**c**) Nucleotide Binding Domain (NBD) with the residues surrounding ADP labelled (F506, E150, I232, G227, Y166, R165, T167, W414, C417). Yellow dashed lines represent hydrogen bonds; (**d**) Steps involved in NLRP3 activation and oligomerization: (1) NEK7 binding, (2) ATP binding and hydrolysis with induced conformational change between the NBD and HD2, (3) dimerization of NLRP3-NEK7 activated complex, (4) oligomerization of the NLRP3-NEK7 complex with ASC and pyrin to give the NLRP3 inflammasome, (5) cleavage of procaspase-1 to mature caspase-1. NLRP3 protein images drawn using PyMOL from PDB: 6NPY.

NLRP3 is composed of the nucleotide binding oligomerization domain (NOD, also called the NACHT domain), a pyrin domain (PYD) at the amino terminal and a leucine-rich repeat (LRR) at the carboxy terminal. The NACHT domain (ATPase domain) of NLRP3 is composed of the nucleotide binding domain (NBD), helical domains HD1 and HD2 and a winged helix domain (WHD) in between HD1 and HD2 (Figure 2a) [7].

The cryo-EM structure of human NLRP3 bound to an artificially engineered dimer of NIMA (never in mitosis gene a)-related kinase 7 (NEK7) (EMD-0476, PDB-6NPY, resolution 3.8 Å) has been reported (Figure 2b) [7]. The serine/threonine kinase NEK7 is important for NLRP3 activation [7] and is shown to bind through its C-terminal lobe to NLRP3. ATP is required for NLRP3 inflammasome activation: ATP binds to the nucleotide binding domain (NBD) of NLRP3 and is hydrolyzed to ADP by the ATPase function (Figure 2c) [18,19]. The energy released from either binding and/or hydrolysis of ATP is proposed to induce a conformationally modified protein, important for NLRP3 inflammasome oligomerization and activation (Figure 2d) [7].

It is of interest to note that two of the five conserved motifs found in NLRP1-14^NACHT^ include Walker A and Walker B [19], which are protein sequences found in ATP-requiring enzymes, initially described by Walker and co-workers [20]. Walker A (P loop), characterized by the sequence G-xxxx-GK-[S/T] (x: Any amino acid), forms part of the ATP binding site responsible for ATPase activity, with the lysine residue interacting with the γ-phosphate through hydrogen-bonding [21,22].

## 5. Role of NEK7 in Inflammasome Activation

NEK7 is a ser/thr kinase that was found to be important for NLRP3 activation (PDB: 6NPY) [7]. To further elucidate the requirement of NEK7 for NLRP3 activation, a knockout experiment was carried out for NEK7 in iBMDMs:mutants prevented nigericin-induced NLRP3 activation whereas wild type NEK7 retained ASC-speck formation, caspase-1 processing and IL-1β release [7,23]. NEK7 was found to bind through its C-terminal lobe to LRR through half of its lobe whilst the other half is associated with the HD2 and NBD regions [7]. NEK7 forms a dimeric complex with NLRP3, proposed to be bound together via a flexible linker [7] (Figure 2b,d).

NEK7, present in low cellular amounts, binds to NEK9 for cell division and is only bound to NLRP3 in the interphase upon exposure of NLRP3 to primary signals, thus NEK7 is a crucial modulator of inflammasome activation [7,24]. It is important to note that NEK7 alone cannot activate NLRP3: ATP needs to be bound to the NBD domain, which phosphorylates S195 of NEK7 allowing NLRP3 to adopt its active conformation for oligomerization. The active conformer occurs when the NACHT domain rotates away from the HBD1 domain as a rigid body, resulting in a diminished point of contact between NEK7 and the NLRP3 NBD domain (Figure 2d) [7]. It should be noted however that NEK7 dependence is not always required: When cells engage a Transforming growth factor-β-Activated Kinase 1 (TAK1)-dependent post-translational priming pathway, NEK7 is no longer required for NLRP3 activation [25].

## 6. Mechanism of Action of NLRP3 Inhibitors: Covalent Modifiers of the NACHT Domain

**Figure 3:**
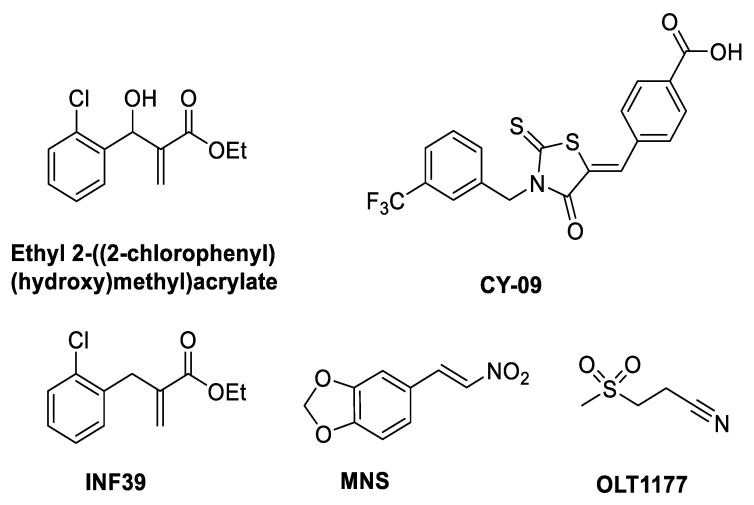
Structures of direct NLRP3 inhibitors that have the potential to be covalent modifiers.

Bertinaria and coworkers led the development of α,β-unsaturated “electrophilic warheads” as inflammasome inhibitors. Hits included ethyl 2-((2-chlorophenyl)(hydroxy)methyl)acrylate (Figure 3) (Table 1), which prevented the pyroptosis of THP-1 cells [26]. Chemically this Michael acceptor reacted covalently with glutathione (as a model thiol) and was also shown to inhibit NLRP3 ATPase activity [26].

CY-09 (Figure 3) (Table 1), a small molecule identified through screening in an in-house bioactive compound library in mouse bone marrow derived macrophages (BMDMs) against NLRP3, has an IC_50_ of 6µM. Furthermore, CY-09 causes inhibition of IL-1β and caspase-1 activity in synovial fluid cells from patients with gout. CY-09 has been proposed to be a direct covalent modifier of the NLRP3 inflammasome, preventing its oligomerization by binding to the ATP-binding site of the NACHT domain [27]. CY-09 has an α,β-unsaturated carbonyl Michael acceptor group which binds covalently with a nucleophile on the Walker A site [27], although this reaction may be reversible (see SI [28]). Computational docking and molecular dynamics simulations on NLRP^NACHT^ protein models with apo and halo ADP-/ATP-Mg^2+^ were used to investigate how CY-09 inhibits ATPase and inhibits NLRP3 activation [27].

**Figure 4:**
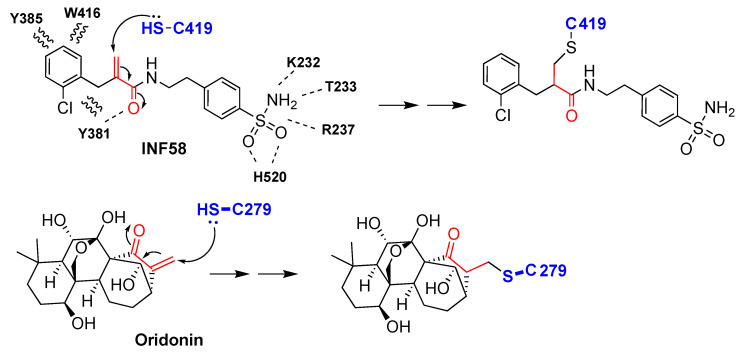
Structures of NLRP3 inhibitors INF58 and oridonin. Covalent bond formation of these inhibitors with cysteine residues in the NACHT domain of NLRP3 [28,31].

NLRP3 inflammasome inhibitors MNS (3,4-methylenedioxy-β-nitrostyrene) (Figure 3) [29], INF39 (ethyl 2-(2-chlorobenzyl)acrylate) (Figure 3) [30], INF58 (Figure 4) [31] and OLT1177 (dapansutrile) (Figure 3) [32] also bind to the ATP binding site. A cysteine residue in the ATP-binding site of the NACHT domain reacts covalently with MNS through a Michael addition. MNS prevents the oligomerization and activation of the NLRP3 inflammasome, inhibiting LPS-induced NLRP3 activation and IL-1β release from mouse BMDMs with an IC_50_ of 2 µM (Table 1) [29]. INF39 was reported as a non-toxic irreversible NLRP3 inflammasome inhibitor with an IC_50_ of 10 µM (Table 1) [30]. INF58 inhibited the ATPase activity of the NLRP3 inflammasome with an IC_50_ of 74 µM, the proposed mechanism being through the covalent reaction of C419 with the Michael acceptor in INF58 (Figure 4) (Table 1) [31]. OLT1177 (dapansutrile) was found to prevent the NLRP3 interaction with ASC through the inhibition of ATPase, resulting in inhibition of IL-1β and IL-18 release from J774 macrophages with an IC_50_ of 1 nM (Table 1) [32].

Oridonin (Figure 4), an electrophilic natural ent-kaurane diterpenoid derivative obtained from *Rabdosia rubescens*, showed anti-inflammatory activity with an IC_50_ of ~0.75 μM. In contrast to the other Michael acceptors which inhibit ATPase activity, oridonin forms a covalent link with C279, blocking the interaction between NLRP3 and NEK7 and preventing a conformational change, thus inhibiting NLRP3 inflammasome assembly and activation (Table 1) [28].

**Table 1:** Direct inhibitors of the NLRP3 ATPase site and their activities.

Inhibitor	% Inhibition at 10 µM /IC_50_	Mechanism of Action
Ethyl 2-((2-chlorophenyl) (hydroxy)methyl)acrylate	75.1 ± 2.6% pyroptosis	Inhibits NLRP3 ATPase [26]
CY-09	IC_50_ = 6 µM	Walker A inhibitor [27]
MNS	IC_50_ = 2 µM	Inhibits NLRP3 ATPase [29]
OLT1177 (dapansutrile)	IC_50_ = 1 nM	ATPase inhibitor blocks NLRP3-ASC interaction [32]
INF39	IC_50_ = 10 µM	Irreversible NLRP3 inflammasome inhibitor [30]
INF58	IC_50_ = 74 µM	ATPase inhibitor [31]
Oridonin	IC_50_ = 0.75 µM	ATPase inhibitor blocks NLRP3-NEK7 interaction [28]
MCC950/CRID3	IC_50_ = 7.5 nM	Walker B inhibitor [33,34,35]

## 7. Mechanism of Action of NLRP3 Inhibitors: MCC950 Binds to Walker B

Walker B, an amino acid chain denoted by hLhhh[D/E] (h: Any hydrophobic residue), is present downstream of the Walker A motif. ATP hydrolysis requires a glutamate residue, which together with aspartate are involved in the formation of co-ordinate bonds with magnesium ions. Aspartate, the first acidic residue in the NLRP3 Walker B site, was found to be within 5Å of ATP-Mg^2+^ [19].

**Figure 5:**
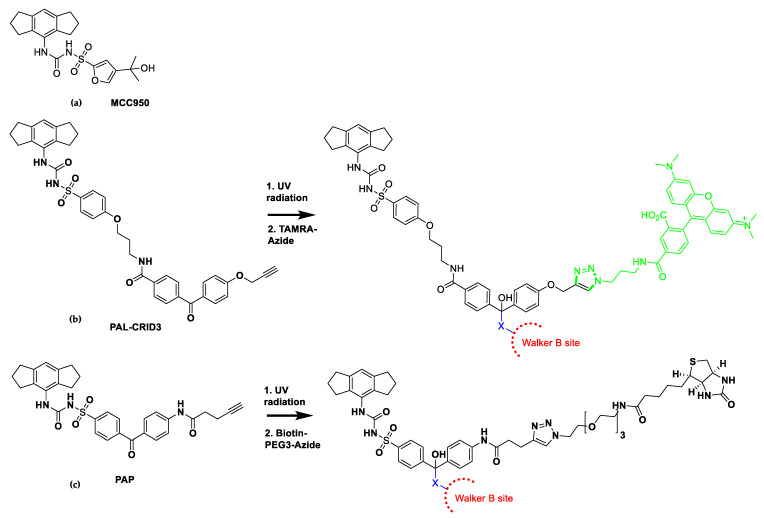
(**a**–**c**) Structure of inflammasome inhibitor MCC950 and its photoaffinity probes PAL-CRID3 and PAP designed to probe the mechanism of action of MCC950; (**b**) In the presence of UV radiation, the benzophenone group in PAL-CRID3 cross-linked using radical chemistry to the NLRP3 protein. The alkyne group of the PAL-CRID3 protein-complex was reacted with TAMRA-azide using click chemistry and the fluorescent protein was isolated by gel electrophoresis; (**c**) In the presence of UV radiation, the benzophenone group in PAP cross-linked using radical chemistry to the NLRP3 protein. The alkyne group of the PAP protein complex was reacted with biotin-PEG3-azide, and the protein isolated using a streptavidin column. Experiments with both photoaffinity probes supported MCC950 binding to the Walker B region of NLRP3 [34,35].

MCC950 (also known as CP-456773 and CRID3) (Figure 5a) is a potent and well-studied inflammasome lead compound, which inhibits NLRP3-dependent ASC oligomerization, with an IC_50_ of 7.5 nM in BMDMs (Table 1) [33]. MCC950 did not compete with ATP upon exposure to wild-type NLRP3ΔLRR, showing that it was unlikely to bind at the Walker A site. The following mechanism of action studies provided evidence that MCC950 binds with high affinity to the Walker B site, and was proposed to form a complex with ATP. Vande Walle and coworkers [34] designed a cell-penetrant photo-affinity label (PAL) probe attached to an MCC950 analogue, PAL-CRID3, which inhibited NLRP3 [34]. Upon UV exposure of PAL-CRID3, the photo-reactive benzophenone undergoes covalent binding to the active site. The alkyne group of PAL-CRID3 reacts with a 5-carboxytetramethylrhodamine (TAMRA) fluorescent reporter using click chemistry, which after cell lysis allows for in-gel fluorescence detection and separation of the covalent MCC950-protein adduct by SDS-PAGE gel electrophoresis, showing direct binding of PAL-CRID3 to NLRP3 (Figure 5b) [34]. Furthermore, Coll and coworkers also designed a photoaffinity probe of MCC950: PAP was covalently cross-linked to its target when activated by UV light, which was then reacted with biotin-PEG3-azide via Cu(1)-catalysed click chemistry and purified using streptavidin magnetic beads (Figure 5c), again supporting binding of MCC950 close to the Walker B site [35].

Tapia-Abellán and coworkers [36] used Bioluminescence Resonance Energy Transfer (BRET) to study the molecular conformation of NLRP3 and showed that MCC950 results in closure of the open active conformation of NLRP3 to an inactive state [36]. Coll and coworkers [35] investigated the protein-ligand interaction formed between MCC950 and the NACHT domain using a drug affinity responsive target stability (DARTS) assay. The principle is based on the protein being resistant to degradation by proteases once it is stabilized by interaction with the inhibitor. MCC950 protected NLRP3 from proteolytic degradation supporting its direct interaction with NLRP3 [35].

In summary, these mechanism of action studies show that MCC950 is proposed to bind non-covalently with high affinity close to the Walker B site on NLRP3. This prevents a structural conformational change of the ATP-binding site keeping the NLRP3 inflammasome in an inactive state, preventing hydrolysis of ATP needed for NLRP3 assembly [34,35,36]. A small clinical study with MCC950 was carried out by Pfizer for rheumatoid arthritis, however increasing concentrations of MCC950 gave rise to an increased risk of liver toxicity, causing the development of MCC950 to cease after phase 1b testing [37]. Attention was then focused by some companies and academia to discover less toxic analogues of MCC950, which are discussed in the next section.

## 8. Clinical Trials and Patents of NLRP3 Inflammasome Inhibitors in the Pharmaceutical Industry Pipeline

Several companies, including Inflazome [4], IFM Therapeutics [3], NodThera [5], Novartis [2], Olatec therapeutics [6] and ZyVersa Therapeutics [38] have small-molecule NLRP3 inflammasome inhibitors that are currently in their drug discovery pipelines in either preclinical or clinical trials. The structures of the compounds have not been disclosed, with the exception of OLT1177 (dapansutrile) from Olatec therapeutics which is in phase 2 trials for oral use in systemic inflammation and treatment of Covid-19 [6,39,40]. Partnering with Novartis, IFM Therapeutics have IFM2427, a systemic-peripheral NLRP3 antagonist in phase 1 clinical trials for the treatment of chronic inflammatory diseases including gout, atherosclerosis and non-alcoholic steatohepatitis [2]. In addition, IFM therapeutics have a gut-directed and a CNS-penetrant NLRP3 antagonist in pre-clinical trials [3].

**Figure 6:**
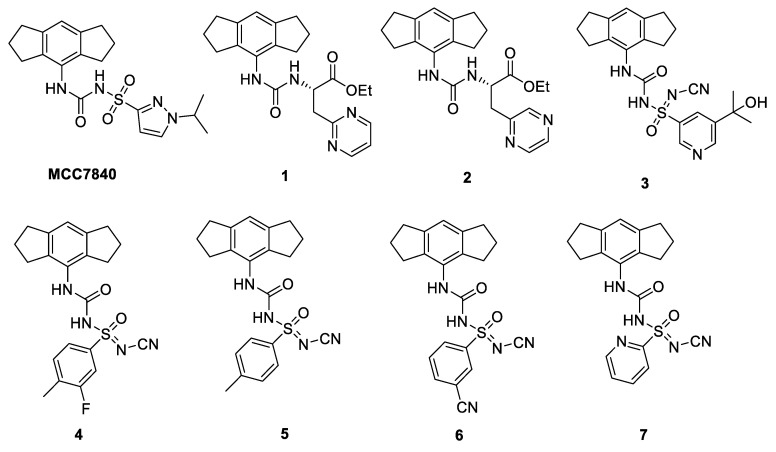
Structures of NLRP3 inhibitors: MCC950 analogues.

Nodthera has been granted patents on NLRP3 inhibitors covering carbamoyl derivatives [41]. NodThera has reported NT-0167, which has shown promising NLRP3 inhibition reducing IL-1β production in preclinical studies and is currently under investigation in healthy volunteers in phase I clinical trials [42]. Potent membrane permeable urea ester prodrugs, inspired by MCC950, have been reported as NLRP3 inhibitors, the most active being 2-[(phenylcarbamoyl)amino]acetates containing 2-pyrimidine (**1**) or 2-pyrazine (**2**) groups (Figure 6), with IC_50s_ of 36 nM and 30 nM for the inhibition of IL-1β in PBMC cells (Table 2). These two compounds were shown to be equipotent to MCC950, however they showed enhanced activity (**1**, 20-fold and **2**, 8-fold) when tested in the whole blood assay when compared with MCC950 [43,44].

Inflazome, a biotech company whose vision is to develop potent, best-in-class, small molecule NLRP3 modulators for the treatment of a broad range of inflammatory diseases, has been granted US and Europe patents for its potent and selective lead NLRP3 inflammasome inhibitors: Somalix (peripheral) is in phase 2 clinical trials and inzomelid (CNS penetrant) has completed phase 1 [4,45,46].

O’Neill and coworkers hold a patent on sulfonylureas, including MCC950 (Figure 5) and MCC7840 (Figure 6) which inhibited NLRP3 with IC_50_ values of <100 nM [47]. The University of Queensland and Cadila have disclosed NLRP3 inhibitors, with the novel substituted sulfoximine compound (**3**) being the most active with a low nM IC_50_ value in THP1 cells (Figure 6) (Table 2) [48]. It has recently been reported that this series of *N*-cyano sulfoximine urea derivatives were highly potent and selective towards NLRP3 inhibition, showing good pharmacokinetic profiles with oral bioavailability. The most active compounds **4**–**7** gave IC_50_ values of 5, 7, 12 and 23 nM, respectively, in the IL-1β inhibition assay in mice (Figure 6) (Table 2) [49].

## 9. Computational Approaches to Design New NLRP3 Inhibitors

### 9.1. Molecular Dynamics

**Figure 7:**
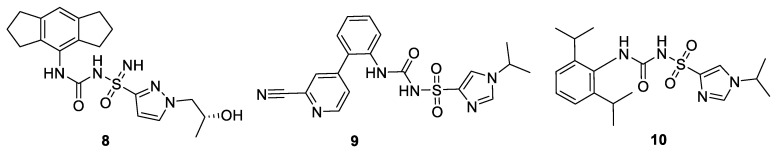
Analogues of MCC950: Patented compounds **8–10** [50].

A homology model of NLRP3 was prepared from the crystal structure of NLRC4 and the FASTA sequence of human NLRP3 (www.uniprot.org). Perricone and coworkers used this homology model in a Molecular Dynamics (MD) study with MCC950 (Figure 5) and its analogues [51]. Detection of all potential druggable binding sites close to the Walker B region were detected by docking MCC950 as a probe, using grid-based and geometry-based algorithm approaches. Docking and MD simulations gave a good RMSD of 1.10 Å with DFT ligand optimization outcomes compared with (Epik) LigPrep tool. The Glide docking tool was used for MCC950 to give extra precision with and without ATP in the NLRP3. A favorable docking score of −8.8 Kcal/mol was obtained when ATP was bound, compared to −6.0 Kcal/mol for the docking pose without ATP. Analogues of MCC950, compounds **8-10** were shown to have IC_50_ of < 1 µM in THP-1 cells [50] (Figure 7) (Table 2) were docked in the same site as MCC950, showing interactions with amino acids P281 and S271 [51].

### 9.2. Virtual Screening (VS) on ER-β Linked to NLRP3 Inflammasome

**Figure 8:**
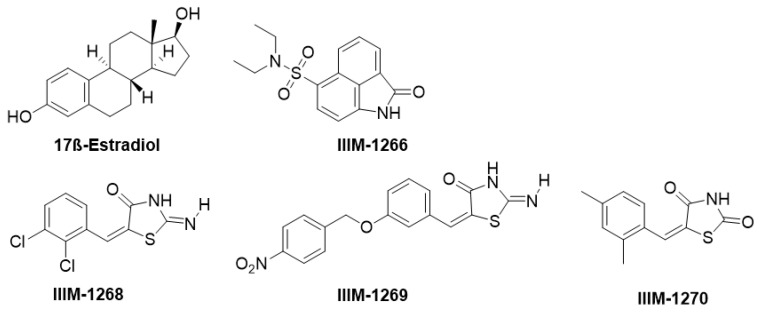
NLRP3 inhibitors identified from the virtual screening of a compound library with the estrogen receptor beta crystal structure [54].

It has been reported that NLRP3 inhibition is linked to estrogen receptor beta (ER-β) upregulation [52]. A virtual screening study was carried out on the ER-β crystal structure (PDB: 5TOA) [53] active site with screening of 20,000 compounds (ChemBridge library). Lead compounds benzo[cd]indol-2-one (IIIM-1266) and benzylidene-thiazolidine-2,4-dione (IIIM-1268, IIIM-1269, IIIM-1270) (Figure 8) were also docked into the ADP-binding site (Walker A/ Walker B) of the cryo-EM structure of NLRP3 (PDB: 6NPY), giving comparable docking scores to MCC950 [54]. IIIM-1266, IIIM-1268, IIIM-1269, and IIIM-1270 showed inhibition of IL-1β release in mouse macrophage (J774A.1) cells of 77.4%, 72.9%, 68.9%, and 74.7% at 10 µM, respectively. The IC_50_ values for IIIM-1268 and IIIM-1270 were 2.3 µM and 3.5 µM [54], although further biology is desirable to both confirm their direct inhibition of the NLRP3 inflammasome and validate its link with ER-β.

### 9.3. Computational Strategies for the Development of NLRP3 Inhibitors by Direct Binding to NLRP3 Pyrin Domain

**Figure 9:**
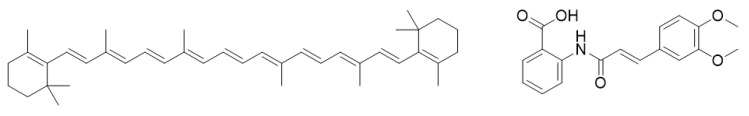
Structures of NLRP3 inhibitors β-carotene (**left**) and tranilast (**right**).

The crystal structure of NLRP3 pyrin domain (PDB: 3QF2) [55] has widened the scope for the discovery of NLRP3 inhibitors. The pyrin domain at the amino terminus of NLRP3 binds to ASC via PYD-PYD interactions. A study reported virtual screening on the NLRP3 PYD domain by utilizing the ZINC database to screen 6 million compounds using Schrodinger A, LigPrep properties along with Glide module in the Schrodinger molecular simulations package. β-Carotene (provitamin A) (Figure 9) was identified from the virtual screen. Using surface plasmon resonance (SPR), an experimental technique used to detect molecular interactions, showed that β-carotene bound directly to the recombinant human pyrin domain of NLRP3 (K_D_ = 3.41 × 10^−6^), blocking interaction of ASC with NLRP3 resulting in inhibition of inflammasome activation. Mutation studies and the BMDM gouty arthritis mouse model activated with MSU were used to further confirm the anti-inflammatory effect of β-carotene [56].

It is interesting to note that tranilast (Figure 9), a drug that has been used clinically for the treatment of a number of inflammatory disorders, inhibits inflammasome activation with an IC_50_ of 10–15 µM [57]. Tranilast has been reported to have a related mechanism of action as β-Carotene, binding to the NACHT domain, inhibiting NLRP3-ASC oligomerization and inflammasome activation [57]. Inhibition of this protein-protein interaction is likely to be exploited in future drug design of inhibitors of the inflammasome.

## 10. Conclusions

The NLRP3 inflammasome has been the focus of drug discovery research due to its involvement in several inflammatory diseases. Inhibitors either have an indirect effect or a direct effect on NLRP3 and in this review there is a focus on those with a direct effect. MCC950, a potent inflammasome inhibitor with an IC_50_ of 7.5 nM, acts directly at or near the Walker B site, whereas CY09 is thought to act directly through covalent modification of the Walker A binding site. The cryo-EM structure of human NLRP3 bound to NEK7 has provided insights on the mechanism of signaling and assembly of the NLRP3 inflammasome, with molecular docking in the ADP-binding site, molecular dynamic simulations, pharmacophore-based screening and virtual screening supporting the discovery of potent NLRP3 inhibitors with appropriate PK properties.

A number of pharmaceutical companies have drug discovery programs to identify novel NLRP3 inflammasome inhibitors, with several compounds currently undergoing clinical trials. The outcome of these trials will of course have a major impact on the future direction of NLRP3 inflammasome research in the pharmaceutical industry. For example, inhibition of the inflammasome comes with a risk of infection, therefore formulations designed for localized or targeted use may be beneficial. In addition, recent studies have shown that post-translational modification of the inflammasome is essential [58,59], for example phosphorylation and ubiquitination, which identifies potential targets for drug discovery.

Although this review focuses only on direct inhibition of the NLRP3 inflammasome, there is significant interest in the inhibition of upstream processes, which again may lessen the risk of infection. For example, our research group has recently shown that chloride channels are important regulators of the inflammasome, with novel urea-based compounds showing promising indirect inhibition of the NLRP3 inflammasome [60,61]. What is certain is that it will be a highly competitive race to discover the “First-in-Class” small molecule clinical NLRP3 inhibitor, which may have the potential to treat a range of therapeutic areas where inflammation is a significant component.

## Figures and Tables

**Table 2:** Activities of MCC950 analogues in the NLRP3 inflammasome.

Inhibitor	IC_50_
MCC7840	<100 nM [47]
1	36 nM [43]
2	30 nM [43]
3	1.26 nM [48]
4	5 nM [49]
5	7 nM [49]
6	12 nM [49]
7	23 nM [49]
8	<1 µM [50]
9	<1 µM [50]
10	<1 µM [50]

## References

[1] Baldwin A.G., Brough D., Freeman S. (2016). Inhibiting the Inflammasome: A Chemical Perspective. J. Med. Chem..

[2] https://www.novartis.com/news/media-releases/novartis-adds-clinical-and-preclinical-anti-inflammatory-programs-portfolio-acquisition-ifm-tre.

[3] https://www.ifmthera.com/pipeline.

[4] https://www.inflazome.com/pipeline.

[5] https://www.nodthera.com.

[6] http://www.olatec.com.

[7] Sharif H., Wang L., Wang W.L., Magupalli V.G., Andreeva L., Qiao Q., Hauenstein A.V., Wu Z., Núñez G., Mao Y. (2019). Structural Mechanism for NEK7-Licensed Activation of NLRP3 Inflammasome. Nature..

[8] Kumar V., Abbas A.K., Aster J.C. (2017). Robbins Basic Pathology.

[9] Lee H.E., Lee J.Y., Yang G., Kang H.C., Cho Y.-Y., Lee H.S., Lee J.Y. (2019). Inhibition of NLRP3 Inflammasome in Tumor Microenvironment Leads to Suppression of Metastatic Potential of Cancer Cells. Sci. Rep..

[10] Lu A., Li H., Niu J., Wu S., Xue G., Yao X., Guo Q., Wan N., Abliz P., Yang G. (2017). Hyperactivation of the NLRP3 Inflammasome in Myeloid Cells Leads to Severe Organ Damage in Experimental Lupus. J. Immunol..

[11] Braga T.T., Forni M.F., Correa-Costa M., Ramos R.N., Barbuto J.A., Branco P., Castoldi A., Hiyane M.I., Davanso M.R., Latz E. (2017). Soluble Uric Acid Activates the NLRP3 Inflammasome. Sci. Rep..

[12] Zheng F., Xing S., Gong Z., Xing Q. (2013). NLRP3 Inflammasomes Show High Expression in Aorta of Patients with Atherosclerosis. Heart Lung Circ..

[13] Kuwar R., Rolfe A., Di L., Xu H., He L., Jiang Y., Zhang S., Sun D. (2019). A Novel Small Molecular NLRP3 Inflammasome Inhibitor Alleviates Neuroinflammatory Response Following Traumatic Brain Injury. J. Neuroinflamm..

[14] Chen I.-Y., Moriyama M., Chang M.-F., Ichinohe T. (2019). Severe Acute Respiratory Syndrome Coronavirus Viroporin 3a Activates the NLRP3 Inflammasome. Front. Microbiol..

[15] Hafner-Bratkovič I., Sušjan P., Lainšček D., Tapia-Abellán A., Cerović K., Kadunc L., Angosto-Bazarra D., Pelegrin P., Jerala R. (2018). NLRP3 Lacking the Leucine-Rich Repeat Domain can be Fully Activated via the Canonical Inflammasome Pathway. Nat. Commun..

[16] Lu A., Magupalli V.G., Ruan J., Yin Q., Atianand M.K., Vos M., Schröder G.F., Fitzgerald K.A., Wu H., Egelman E.H. (2014). Unified Polymerization Mechanism for the Assembly of ASC-Dependent Inflammasomes. Cell.

[17] Dubois H., Sorgeloos F., Sarvestani S.T., Martens L., Saeys Y., Mackenzie J.M., Lamkanfi M., van Loo G., Goodfellow I., Wullaert A. (2019). NLRP3 Inflammasome Activation and Gasdermin D-Driven Pyroptosis are Immunopathogenic upon Gastrointestinal Norovirus Infection. PLoS Pathog..

[18] Duncan J.A., Bergstralh D.T., Wang Y., Willingham S.B., Ye Z., Zimmermann A.G., Ting J.P.-Y. (2007). Cryopyrin/NALP3 Binds ATP/dATP, is an ATPase, and Requires ATP Binding to Mediate Inflammatory Signaling. Proc. Natl. Acad. Sci. USA.

[19] Maharana J., Panda D., De S. (2018). Deciphering the ATP-Binding Mechanism(s) in NLRP-NACHT 3D Models using Structural Bioinformatics Approaches. PLoS ONE.

[20] Walker J.E., Saraste M., Runswick M.J., Gay N.J. (1982). Distantly Related Sequences in the Alpha- and Beta-Subunits of ATP Synthase, Myosin, Kinases and other ATP-Requiring Enzymes and a Common Nucleotide Binding Fold. EMBO J..

[21] Neuwald A.F., Aravind L., Spouge J.L., Koonin E.V. (1999). AAA+: A Class of Chaperone-Like ATPases Associated with the Assembly, Operation, and Disassembly of Protein Complexes. Genome Res..

[22] Ye Z., Lich J.D., Moore C.B., Duncan J.A., Williams K.L., Ting J.P.-Y. (2008). ATP Binding by Monarch-1/NLRP12 is Critical for its Inhibitory Function. Mol. Cell. Biol..

[23] Schmid-Burgk J.L., Chauhan D., Schmidt T., Ebert T.S., Reinhardt J., Endl E., Hornung V. (2016). A Genome-wide CRISPR Screen Identifies NEK7 as an Essential Component of NLRP3 Inflammasome Activation. J. Biol. Chem..

[24] He Y., Zeng M.Y., Yang D., Motro B., Núñez G. (2016). NEK7 is an Essential Mediator of NLRP3 Activation Downstream of Potassium Efflux. Nature.

[25] Schmacke N.A., Gaidt M.M., Szymanska I., O’Duill F., Stafford C.A., Chauhan D., Fröhlich A.L., Nagl D., Pinci F., Schmid-Burgk J.L. (2019). Priming Enables a NEK7-Independent Route of NLRP3 Activation. bioRxiv.

[26] Cocco M., Garella D., Di Stilo A., Borretto E., Stevanato L., Giorgis M., Marini E., Fantozzi R., Miglio G., Bertinaria M. (2014). Electrophilic Warhead-Based Design of Compounds Preventing NLRP3 Inflammasome-Dependent Pyroptosis. J. Med. Chem..

[27] Jiang H., He H., Chen Y., Huang W., Cheng J., Ye J., Wang A., Tao J., Wang C., Liu Q. (2017). Identification of a Selective and Direct NLRP3 Inhibitor to Treat Inflammatory Disorders. J. Exp. Med..

[28] He H., Jiang H., Chen Y., Ye J., Wang A., Wang C., Liu Q., Liang G., Deng X., Jiang W. (2018). Oridonin is a Covalent NLRP3 Inhibitor with Strong Anti-Inflammasome Activity. Nat. Commun..

[29] He Y., Varadarajan S., Muñoz-Planillo R., Burberry A., Nakamura Y., Núñez G. (2014). 3,4-Methylenedioxy-β-nitrostyrene Inhibits NLRP3 Inflammasome Activation by Blocking Assembly of the Inflammasome. J. Biol. Chem..

[30] Cocco M., Pellegrini C., Martínez-Banaclocha H., Giorgis M., Marini E., Costale A., Miglio G., Fornai M., Antonioli L., López-Castejón G. (2017). Development of an Acrylate Derivative Targeting the NLRP3 Inflammasome for the Treatment of Inflammatory Bowel Disease. J. Med. Chem..

[31] Cocco M., Miglio G., Giorgis M., Garella D., Marini E., Costale A., Regazzoni L., Vistoli G., Orioli M., Massulaha-Ahmed R. (2016). Design, Synthesis, and Evaluation of Acrylamide Derivatives as Direct NLRP3 Inflammasome Inhibitors. ChemMedChem.

[32] Marchetti C., Swartzwelter B., Gamboni F., Neff C.P., Richter K., Azam T., Carta S., Tengesdal I., Nemkov T., D’Alessandro A. (2018). OLT1177, a β-Sulfonyl Nitrile Compound, Safe in Humans, Inhibits the NLRP3 Inflammasome and Reverses the Metabolic Cost of Inflammation. Proc. Natl. Acad. Sci. USA.

[33] Coll R.C., Robertson A.A.B., Chae J.J., Higgins S.C., Muñoz-Planillo R., Inserra M.C., Vetter I., Dungan L.S., Monks B.G., Stutz A. (2015). A Small-Molecule Inhibitor of the NLRP3 Inflammasome for the Treatment of Inflammatory Diseases. Nat. Med..

[34] Vande Walle L., Stowe I.B., Šácha P., Lee B.L., Demon D., Fossoul A., Van Hauwermeiren F., Saavedra P.H.V., Šimon P., Šubrt V. (2019). MCC950/CRID3 Potently Targets the NACHT Domain of Wild-Type NLRP3 but not Disease-Associated Mutants for Inflammasome Inhibition. PLoS Biol..

[35] Coll R.C., Hill J.R., Day C.J., Zamoshnikova A., Boucher D., Massey N.L., Chitty J.L., Fraser J.A., Jennings M.P., Robertson A.A.B. (2019). MCC950 Directly Targets the NLRP3 ATP-Hydrolysis Motif for Inflammasome Inhibition. Nat. Chem. Biol..

[36] Tapia-Abellán A., Angosto-Bazarra D., Martínez-Banaclocha H., Torre-Minguela C.D., Cerón-Carrasco J.P., Pérez-Sánchez H., Arostegui J.I., Pelegrin P. (2019). MCC950 Closes the Active Conformation of NLRP3 to an Inactive State. Nat. Chem. Biol..

[37] Coss R. (2020). Could an NLRP3 Inhibitor Be the One Drug to Conquer Common Diseases?. C EN Glob. Enterp..

[38] https://www.zyversa.com.

[39] Sánchez-Fernández A., Skouras D.B., Dinarello C.A., López-Vales R. (2019). OLT1177 (Dapansutrile), a Selective NLRP3 Inflammasome Inhibitor, Ameliorates Experimental Autoimmune Encephalomyelitis Pathogenesis. Front. Immunol..

[40] https://www.businesswire.com/news/home/20201027005190/en/Olatec-Therapeutics-Enrolls-its-First-Patients-in-a-Phase-2-Clinical-Trial-in-COVID-19-with-its-Selective-NLRP3-Inhibitor-Oral-Dapansutrile.

[41] Bock M.G., Watt A.P., Porter R.A., Harrison D., Boutard N.F.P., Levenets O., Fabritius C.-H.R.Y., Topolnicki G.W. (2019). Selective Inhibitors of NLRP3 Inflammasome.

[42] https://www.nodthera.com/nodthera-announces-close-of-55-million-series-b-financing/.

[43] Harrison D., Boutard N., Brzozka K., Bugaj M., Chmielewski S., Cierpich A., Doedens J.R., Fabritius C.-H.R.Y., Gabel C.A., Galezowski M. (2020). Discovery of a Series of Ester-substituted NLRP3 Inflammasome Inhibitors. Bioorg. Med. Chem. Lett..

[44] Harrison D., Watt A.P., Boutard N., Fabritius C.-H., Galezowski M., Kowalczyk P., Levents O., Woyciechowski J. (2018). Chemical Compounds.

[45] https://www.businesswire.com/news/home/20200226006027/en/Inflazome’s-Somalix-Demonstrates-Positive-Safety-Tolerability-Pharmacodynamic.

[46] https://www.businesswire.com/news/home/20200326005102/en/Inzomelid-completes-Phase-studies-shows-positive-results.

[47] O’Neill L., Coll R., Cooper M., Robertson A., Schroder K. (2016). Sulfonylureas and Related Compounds and Use of Same.

[48] Sharma R., Iyer P., Agarwal S. (2018). Novel Substituted Sulfoximine Compounds.

[49] Agarwal S., Sasane S., Shah H.A., Pethani J.P., Deshmukh P., Vyas V., Iyer P., Bhavsar H., Viswanathan K., Bandyopadhyay D. (2020). Discovery of *N*-Cyano-sulfoximineurea Derivatives as Potent and Orally Bioavailable NLRP3 Inflammasome Inhibitors. ACS Med. Chem. Lett..

[50] Cooper M., Miller D., Macleod A., Van Wiltenberg J., Thom S., ST-Gallay S., Shannon J. (2019). Sulfonylureas and Sulfonylthioureas as NLRP3 Inhibitors.

[51] Mekni N., De Rosa M., Cipollina C., Gulotta M.R., De Simone G., Lombino J., Padova A., Perricone U. (2019). In Silico Insights towards the Identification of NLRP3 Druggable Hot Spots. Int. J. Mol. Sci..

[52] Shi J., Zhao W., Ying H., Zhang Y., Du J., Chen S., Li J., Shen B. (2018). Estradiol Inhibits NLRP3 Inflammasome in Fibroblast-Like Synoviocytes Activated by Lipopolysaccharide and Adenosine Triphosphate. Int. J. Rheum. Dis..

[53] Souza P.C.T., Textor L.C., Melo D.C., Nascimento A.S., Skaf M.S., Polikarpov I. (2017). An Alternative Conformation of ERβ Bound to Estradiol Reveals H12 in a Stable Antagonist Position. Sci. Rep..

[54] Abdullaha M., Ali M., Kour D., Kumar A., Bharate S.B. (2020). Discovery of Benzo[cd]indol-2-one and Benzylidene-Thiazolidine-2,4-Dione as New Classes of NLRP3 Inflammasome Inhibitors *via* ER-β Structure Based Virtual Screening. Bioorg. Chem..

[55] Bae J.Y., Park H.H. (2011). Crystal Structure of NALP3 Protein Pyrin Domain (PYD) and its Implications in Inflammasome Assembly. J. Biol. Chem..

[56] Yang G., Lee H.E., Moon S.-J., Ko K.M., Koh J.H., Seok J.K., Min J.-K., Heo T.-H., Kang H.C., Cho Y.-Y. (2020). Direct Binding to NLRP3 Pyrin Domain as a Novel Strategy to Prevent NLRP3-Driven Inflammation and Gouty Arthritis. Arthritis Rheumatol..

[57] Huang Y., Jiang H., Chen Y., Wang X., Yang Y., Tao J., Deng X., Liang G., Zhang H., Jiang W. (2018). Tranilast Directly Targets NLRP3 to Treat Inflammasome-Driven Diseases. EMBO Mol. Med..

[58] Seoane P.I., Lee B., Hoyle C., Yu S., Lopez-Castejon G., Lowe M., Brough D. (2020). The NLRP3-Inflammasome as a Sensor of Organelle Dysfunction. J. Cell. Biol..

[59] Shim D.-W., Lee K.-H. (2018). Posttranslational Regulation of the NLR Family Pyrin Domain-Containing 3 Inflammasome. Front. Immunol..

[60] Green J., Swanton T., Morris L., El-Sharkawy L., Cook J., Yu S., Beswick J., Adamson A., Humphreys N., Bryce R. (2020). LRRC8A is Essential for Hypotonicity-, but not for DAMP-Induced NLRP3 Inflammasome Activation. eLife.

[61] Swanton T., Beswick J.A., Hammadi H., Morris L., Williams D., de Cesco S., El-Sharkawy L., Yu S., Green J., Davis J.B. (2020). Selective Inhibition of the K^+^ Efflux Sensitive NLRP3 Pathway by Cl^−^ Channel Modulation. Chem. Sci..

